# How to measure, report and verify soil carbon change to realize the potential of soil carbon sequestration for atmospheric greenhouse gas removal

**DOI:** 10.1111/gcb.14815

**Published:** 2019-10-06

**Authors:** Pete Smith, Jean‐Francois Soussana, Denis Angers, Louis Schipper, Claire Chenu, Daniel P. Rasse, Niels H. Batjes, Fenny van Egmond, Stephen McNeill, Matthias Kuhnert, Cristina Arias‐Navarro, Jorgen E. Olesen, Ngonidzashe Chirinda, Dario Fornara, Eva Wollenberg, Jorge Álvaro‐Fuentes, Alberto Sanz‐Cobena, Katja Klumpp

**Affiliations:** ^1^ Institute of Biological & Environmental Sciences University of Aberdeen Aberdeen UK; ^2^ INRA Paris Cedex 07 France; ^3^ Agriculture and Agri‐Food Canada Quebec QC Canada; ^4^ Environmental Research Institute University of Waikato Hamilton New Zealand; ^5^ INRA, AgroParisTech. Thiverval‐Grignon France; ^6^ Norwegian Institute of Bioeconomy Research (NIBIO) Ås Norway; ^7^ ISRIC – World Soil Information Wageningen The Netherlands; ^8^ Manaaki Whenua – Landcare Research Lincoln New Zealand; ^9^ Department of Agroecology Aarhus University Tjele Denmark; ^10^ International Center for Tropical Agriculture (CIAT) Cali Colombia; ^11^ Agri‐Food and Biosciences Institute Belfast UK; ^12^ CGIAR CCAFS Programme University of Vermont (UVM) Burlington VT USA; ^13^ Soil and Water Department Spanish National Research Council (CSIC) Zaragoza Spain; ^14^ Research Center for the Management of Environmental and Agricultural Risks (CEIGRAM) Universidad Politécnica de Madrid Madrid Spain; ^15^ INRA VetAgro‐Sup UCA Clermont Ferrand France

**Keywords:** measurement, monitoring, MRV, reporting, soil organic carbon, soil organic matter, verification

## Abstract

There is growing international interest in better managing soils to increase soil organic carbon (SOC) content to contribute to climate change mitigation, to enhance resilience to climate change and to underpin food security, through initiatives such as international ‘4p1000’ initiative and the FAO's Global assessment of SOC sequestration potential (GSOCseq) programme. Since SOC content of soils cannot be easily measured, a key barrier to implementing programmes to increase SOC at large scale, is the need for credible and reliable measurement/monitoring, reporting and verification (MRV) platforms, both for national reporting and for emissions trading. Without such platforms, investments could be considered risky. In this paper, we review methods and challenges of measuring SOC change directly in soils, before examining some recent novel developments that show promise for quantifying SOC. We describe how repeat soil surveys are used to estimate changes in SOC over time, and how long‐term experiments and space‐for‐time substitution sites can serve as sources of knowledge and can be used to test models, and as potential benchmark sites in global frameworks to estimate SOC change. We briefly consider models that can be used to simulate and project change in SOC and examine the MRV platforms for SOC change already in use in various countries/regions. In the final section, we bring together the various components described in this review, to describe a new vision for a global framework for MRV of SOC change, to support national and international initiatives seeking to effect change in the way we manage our soils.

## Graphical abstract

Since soil organic carbon (SOC) changes are difficult to measure, a key barrier to implementing programmes to increase SOC is the need for credible and reliable measurement/monitoring, reporting and verification (MRV) platforms. We review methods for measuring SOC change directly in soils, examine novel developments for quantifying SOC change and describe how surveys, long‐term experiments and chronosequences can be used for testing models and as benchmark sites in global frameworks to estimate SOC change. We review MRV platforms for soil organic carbon change already in use and describe a new vision for a global framework for MRV of SOC change.

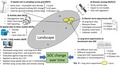

## 1 INTRODUCTION

Soil organic carbon (SOC) represents a stock of around 1,500–2,400 Gt C (~5500–8800 Gt CO_2_) in the top metre of soils globally (Batjes, 1996; Sanderman, Hengl, & Fiske, 2017). The lower estimate in the range is approximately three times the stock of carbon (C) in vegetation and twice the stock of C in the atmosphere (Smith, 2012). Small changes in C stocks can therefore have significant impacts on the atmosphere and climate change. Since the onset of agriculture around 8,000 years ago (Ruddiman, 2005), soils have lost around 140–150 Gt C (~510–550 Gt CO_2_; Sanderman et al., 2017) through cultivation. It is known that best management practices can restore some at least some of this lost carbon (Lal et al., 2018), so it has been suggested that soil C sequestration could be a significant greenhouse gas (GHG) removal strategy (also called negative emission technology, or carbon dioxide removal option; Smith, 2016). Global estimates of soil C sequestration potential vary considerably, but a recent systematic review by Fuss et al. (2018) suggests an annual technical potential of 2–5 Gt CO_2_/year. Estimates of economic potentials are at the lower end of this range (Smith, 2016; Smith et al., 2008).

An incomplete understanding on how SOC changes are influenced by climate, land use, management and edaphic factors (Stockmann et al., 2013), adds complexity to designing appropriate monitoring, reporting and verification (MRV) platforms. For instance, process‐level knowledge on how these variables influence changes in C stocks and fluxes remains incomplete (Bispo et al., 2017). Furthermore, the reversibility of C sequestration, when practices that retain C are not maintained, or due to climate variability or climate change, increases uncertainty in the time frames needed to monitor SOC enhancement activities (Rumpel et al., 2019). In addition, the large background stocks, inherent spatial and temporal variability and slow soil C gains make the detection of short‐term changes (e.g. 3–5 years) in SOC stocks and the design of reliable, cost‐effective and easy to apply MRV platforms challenging (Post, Izaurralde, Mann, & Bliss, 1999).

Smith et al. (2012) described a framework, building on available models, data sets and knowledge, to quantify the impacts of land use and management change on soil carbon. That paper concluded by presenting a future vision for a global framework to assess soil carbon change, based on a combination of mathematical models, spatial data to drive the models, short‐ and long‐term data to evaluate the models, and a network of benchmarking sites to verify estimated changes. Here, we review the new knowledge since then, and further develop this vision in the light of the need to provide credible and robust MRV capabilities to support the growing International and National initiatives to increase SOC, such as the International ‘4p1000’ initiative (Chabbi et al., 2017; Rumpel et al., 2018, 2019).

We focus on methods to *measure* and/or *estimate* SOC change, but these measurement/estimation methods also form the basis of how changes in SOC can be *monitored* and *reported* at plot to national (and even global) scales, and how reported changes could be *verified*. We begin by reviewing the methods and challenges of measuring SOC change directly in soils (Section 2), before examining some recent developments that show promise for quantifying SOC stocks (and therefore change) using flux measurements, non‐destructive field‐based spectroscopic methods and the possibility in future of estimating SOC change through earth observation/remote sensing (Section 3). We then review how repeat soil surveys are used to estimate territorial changes in SOC over time (Section 4), and how long‐term experiments and space‐for‐time substitution sites can serve as sources of knowledge and can be used to testing models, and as potential benchmark sites in global platforms to estimate SOC change (Section 5). Section 6 summarizes recent reviews on models available for simulating and predicting change in SOC, after which Section 7 describes MRV platforms for SOC change already in use in various countries/regions. We finish the review (Section 8) by describing a new vision for a global framework for MRV of SOC change to support national and international initiatives.

## 2 DIRECT MEASUREMENT OF SOC STOCK CHANGES

Accurate estimates of SOC stocks rely strongly on baseline SOC values, which are determined by physical sampling and soil C content measurements. This approach traditionally involves the quantification of (a) fine earth (<2 mm) and coarse mineral (>2 mm) fractions of the soil; (b) organic carbon (OC) concentration (%) of the fine earth fraction; and (c) soil bulk density or fine earth mass (FAO, 2019a). In some instances, such as grasslands or forest soils, it may be of interest to quantify and account for the coarse fraction of belowground OC (FAO, 2019a). The challenge remains to accurately estimate the rock content of sampled soils, which can significantly affect soil bulk density (Page‐Dumroese, Jurgensen, Brown, & Mroz, 1999; Poeplau, Vos, & Don, 2017; Throop, Archer, Monger, & Waltman, 2012). Changes in management that influence carbon content also affect the bulk density of the soil (Haynes & Naidu, 1998), and thereby the amount of soil that is sampled within a given sampling depth. It is therefore recommended to use an ‘equivalent mass basis’ approach when comparing SOC stocks across land uses and different management regimes (Ellert & Bettany, 1995; Upson, Burgess, & Morison, 2016; Wendt & Hauser, 2013).

Direct measurements also rely on appropriate study designs and sampling protocols to deal with high spatial variability of SOC stocks (Minasny et al., 2017). To reduce potential sources of error in SOC stock estimation and minimize the minimum detectable difference (i.e. the smallest difference in SOC stock that can be detected as statistically significant between two sampling periods; FAO, 2019a), a large number of soil samples is often required (Garten & Wullschleger, 1999; Vanguelova et al., 2016). Sufficient sampling depth is a crucial factor for properly evaluating changes in soil C content (IPCC recommends a minimum depth of 30 cm). Several long‐term agronomy experiments suffer from an increase in ploughing depth during more recent decades, as agricultural machinery became more powerful. Insufficient information on historical sampling depth can also add uncertainty to the results.

Several methods for increasing soil C content require deeper sampling for confirming the expected effect. The positive effect of no‐till on soil C content measured in the surface soil may not be apparent when measuring to 60 cm depth (Angers & Eriksen‐Hamel, 2008; Blanco‐Canqui & Lal, 2008). Crops with deep root phenotypes are considered a promising method to increase C sequestration in soils (Paustian et al., 2016), though demonstrating their effect requires deep soil sampling. Deeper soil sampling (100 cm) is recommended (FAO, 2019a), but often requires specific machinery and is costly.

Costs associated with collecting, processing and storing soil samples and C content measurements using, for example, common dry combustion methods (Nelson & Sommers, 1996) can make large‐scale direct measurements of soil SOC stocks prohibitively expensive. It was estimated that to detect meaningful changes in soil C stocks across forest ecosystems in Finland (i.e. 3,000 plots at the national scale) might cost 4 million Euro for one sampling campaign (e.g. baseline measurement from 1 year) and then again for the following sampling interval (e.g. 10 years later; Mäkipää, Häkkinen, Muukkonen, & Peltoniemi, 2008). Thus, there is the need to evaluate these costs against the value of soil C sequestered (Mäkipää et al., 2008; Smith, 2004b) and search for trade‐offs between costs involved and alternative SOC estimation methods including different modelling approaches.

A combination of direct measurements (at the plot scale) and modelling (at larger spatial scales) can greatly help defining the efficacy of different land management practices in enhancing soil C sequestration and has been used for estimating soil C change in national GHG inventory platforms (e.g. VandenBygaart et al., 2008). It is, therefore, crucial to evaluate the cost‐effectiveness of measuring and sequestering C across different land uses and socio‐economic conditions (Alexander, Paustian, Smith, & Moran, 2015).

## 3 NOVEL METHODS OF MEASURING SOC CHANGE

### 3.1 Inferring SOC stock changes from flux measurements

An alternative to repeated measurements is to draw up a full carbon budget. This indirect approach accounts for the initial uptake of carbon through photosynthesis (gross primary production), its subsequent partial losses through respiration (soil, plant and litter) to give net ecosystem exchange (NEE) or net ecosystem production and further C inputs (organic fertilization) and outputs (harvest) to and from the system (see Smith, Lanigan, et al., 2010; Soussana, Tallec, & Blanfort, 2010). The measurements of the net balance of C fluxes exchanged (i.e. estimating NEE) can be achieved by chamber measurements or by the eddy covariance (EC) method (e.g. Baldocchi, 2003). During recent decades, estimates of C sequestration from flux measurements have been reported to be comparatively uncertain due to (a) necessary assumptions associated with data processing (e.g. footprint, spectral corrections, i.e. Aubinet, Vesala, & Papale, 2012); the fact that (b) this method is a point‐in‐space measurement; and (c) net changes in soil C pools are relatively small compared to C stored in biomass and litter when measured over short time periods (i.e. <5 years).

**Figure 1:**
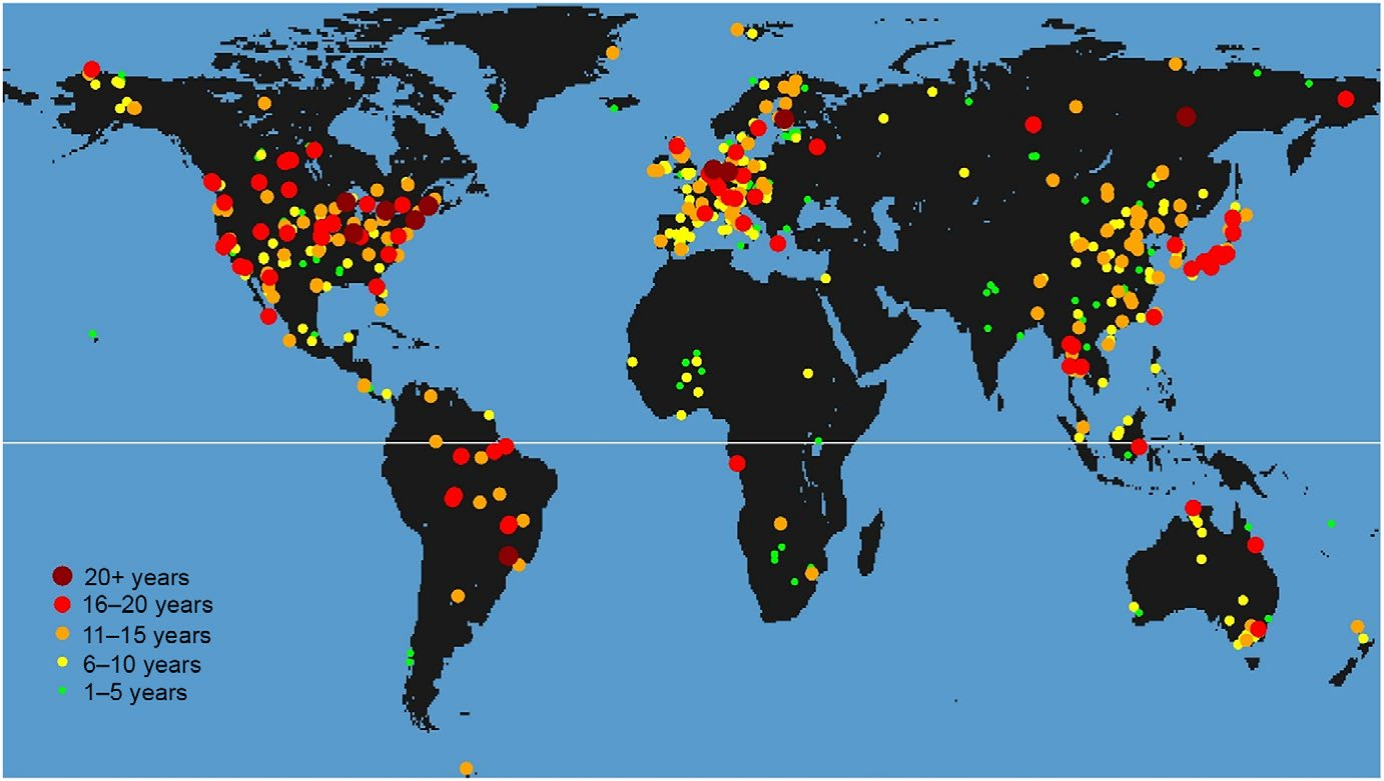
Map of flux towers and available time series worldwide *Source*: Fluxnet, 2019

Despite this, recent developments in instrumentation (analyser performance and set‐ups, e.g. Rebmann et al., 2018), data acquisition and processing (i.e. data loggers, software, QA/QC checks) have greatly improved the reliability of estimates (e.g. Fratini & Mauder, 2014). Furthermore, harmonized networks of long‐term observation sites, created to provide access to standardized data and to quantify the effectiveness of carbon sequestration and/or GHG emission at European (Integrated Carbon Observation System, ICOS; Franz et al., 2018) and global scale (FLUXNET global network, e.g. Baldocchi, Housen, & Reichstein, 2018; Figure 1), have greatly reduced uncertainties in flux and supplementary measurements. Moreover, ongoing analyses on peculiarities of flux measurement likely to increase uncertainties in flux measurements, such as integration of (moving) point sources, that is, grazing animals (Felber, Münger, Neftel, & Ammann, 2015; Gourlez de la Motte et al., 2019), ditches (Nugent, Strachan, Strack, Roulet, & Rochefort, 2018) and fallow periods, have been studied thoroughly and have allowed routine data analyses to be updated (e.g. Sabbatini et al., 2018).

Concerning the comparison between C sequestration determined via the EC technique (i.e. full C balance) and soil C stock changes, some studies have shown poor agreement (Jones et al., 2017), but a number of studies have shown comparable estimates, when applied for time frames >10 year and with soil data including at least both top and medium soil depths (i.e. 0–60 cm; e.g. *grassland*: Leifeld, Ammann, Neftel, & Fuhrer, 2011; Skinner & Dell, 2014; Stahl et al., 2017; *cropland*: Emmel et al., 2018; Hoffmann et al., 2017; *for* *est*: Ferster, Trofymow, Coops, Chen, & Black, 2015). Coupling of EC with soil C stock change studies has become a favoured approach to understand both short‐ and long‐term effects of principal drivers (e.g. management, climate) on ecosystem functioning (i.e. Eugster & Merbold, 2015), *in natura* measurement and modelling approaches (e.g. Beer et al., 2010; Besnard et al., 2018; Williams et al., 2009).

### 3.2 Spectral methods for measuring SOC stocks

New spectral methods for measuring SOC concentration and stocks are rapidly becoming available for direct point measurements in field and in the lab, but also for measurement of patterns at larger scales across landscapes and regions. Each comes with a specific associated accuracy and cost (Bellon‐Maurel & McBratney, 2011; England & Viscarra Rossel, 2018; Nayak et al., 2019). A smart combination of these and more traditional methods can either bring down costs (Nocita et al., 2015), provide more exhaustive spatial patterns of SOC stocks (Aitkenhead, 2017; Rosero‐Vlasova, Vlassova, Pérez‐Cabello, Montorio, & Nadal‐Romero, 2019) or provide indications for change in stocks (Li et al., 2018; Zhao, Ye, Li, Yu, & Mcclellan, 2016).

The methods for measuring SOC concentration mainly rely on the reflectance of light on soil in the infrared region. The organic bonds and minerals in the soil absorb light at specific wavelengths, resulting is a soil content‐specific absorbance or reflectance spectrum. This spectrum is measured with high level of spectral detail (hyperspectral, often in the lab) or limited level of detail in wider bands (multispectral, often from satellites or cheaper field instruments). Using a statistical model based on a spectral library, the soil carbon percentage can be predicted from spectral measurements of the unknown samples. The spectral library is derived from samples on which soil properties have been determined by traditional laboratory methods, such as dry combustion, alongside reflectance measurements. Relevant wavelengths for soil and SOC are mainly in the mid‐ (4,000–600 cm^−1^) and the near‐ or short‐wave infrared region (2,000–2,500 nm). Other key soil properties can also be simultaneously determined if present in the spectral libraries, including fractions of OC and vulnerability of soil carbon to loss (Baldock, Beare, Curtin, & Hawke, 2018; Baldock, Hawke, Sanderman, & Macdonald, 2013), soil texture, pH and others (Stenberg, Viscarra Rossel, Mouazen, & Wetterlind, 2010), which can be used to inform modelling approaches. Partial least squares regression (PLSR) is a statistical method that is currently most widely used to predict soil properties from spectra. These machine learning approaches (e.g. Cubist, Random Forests, Support Vector [regression] Machines and others) are rapidly developing, and new techniques are becoming available, currently referred to as deep learning (Padarian, Minasny, & Mcbratney, 2019) and memory based learning (Dangal, Sanderman, Wills, & Ramirez‐Lopez, 2019; Ramirez‐Lopez et al., 2013). These techniques, such as locally weighted PLSR, use local calibrations based on spectrally similar subsets of a spectral library. This will likely lead to considerable improvement, reducing the prediction errors. This does not resolve the inherent laboratory measurement uncertainties associated with both reference and spectral data.

Standardization of reference laboratory methods, spectral measurements and soil data exchange to some extent negates these issues, and they are addressed in several international co‐operations, one of which is Pillar 5 of the Global Soil Partnership (GSP, 2017). If standardization and calibration transfer challenges can be solved, combining spectral libraries can provide a vast data resource for not only local but also more regional and global SOC analyses (England & Viscarra Rossel, 2018; Viscarra Rossel, Behrens, et al., 2016; Viscarra Rossel, Brus, Lobsey, Shi, & Mclachlan, 2016).

Laboratory costs could be reduced by using Fourier transform mid‐infrared (MIR) diffuse reflectance spectroscopy for estimation of total carbon, OC, clay content and sand fraction (Viscarra Rossel, Walvoort, Mcbratney, Janik, & Skjemstad, 2006; Wijewardane, Ge, Wills, & Libohova, 2018). Several commercial laboratories use near‐infrared (NIR) for this purpose but once a sufficient spectral library or calibration set is compiled, MIR outperforms NIR (Reeves, 2010; Viscarra Rossel et al., 2006; Vohland, Ludwig, Thiele‐Bruhn, & Ludwig, 2014). In such studies or applications, bigger libraries are spiked or subselected to build local (spectral or geographical) prediction models using machine learning techniques (Janik, Skjemstad, Shepherd, & Spouncer, 2007). Sample preparation is very simple (dry, sieve to <2 mm, fine grind (Soil Survey Staff, 2014) and after a library is built, the measurements are fast and inexpensive, and can assess all of the listed properties at the same time (Nocita et al., 2015).

These spectral libraries can also be used to calibrate field spectrometers, although accuracy will often be lower, mostly due to moisture and surface roughness of the soil. Higher cost in situ systems are available for both NIR and MIR (Dhawale et al., 2015; Hutengs, Ludwig, Jung, Eisele, & Vohland, 2018). Alternatives are cheap in‐field NIR spectrometers for point measurements (Tang, Jones, & Minasny, 2019) which tend to have low(er) accuracies due to hardware constraints and which may have bias. On‐the‐go systems with 2–5 wavelengths are on the market as well as penetrometers with visible and near‐infrared reflectance spectroscopy (VNIR), which also provide a measure for penetration resistance or compacted soil (Ackerson, Morgan, & Ge, 2017; Al‐Asadi & Mouazen, 2018; Poggio, Brown, & Bricklemyer, 2017; Wetterlind, Piikki, Stenberg, & Söderström, 2015). A final possibility is a core sampler which measures the extracted soil core in field with VNIR and active gamma radiation for (total) bulk density (Lobsey & Viscarra Rossel, 2016).

An important property for calculating SOC stocks is soil bulk density which is difficult to measure accurately in field (Bellon‐Maurel & McBratney, 2011). A method used in a number of set‐ups is gamma attenuation. This can be measured on the extracted soil core (England & Viscarra Rossel, 2018; Lobsey & Viscarra Rossel, 2016) or directly in the soil (Jacobs, Eelkema, Limburg, & Winterwerp, 2009). With this technique, the attenuation by matter of gamma radiation originating from a small radioactive source is measured over a known volume between source and detector. The matter in this case consists of both soil and moisture. The volume is simulated using Monte Carlo simulations. This provides a measure of dry bulk density after correction for moisture content as measured for instance with a time domain reflectometry (Jacobs et al., 2009) or VNIR (Lobsey & Viscarra Rossel, 2016).

The benefit of these techniques is the possibility to acquire more samples and/or more in‐field measurements, allowing a user to address the potential of carbon sequestration of the soil adequately. Some of these techniques are most suitable for describing the spatial distribution of soil carbon, while others are suitable for quantitative estimates or monitoring (in time, allowing the impacts of management on soil carbon to be detected). Choices can be made based on cost and required accuracy of the purpose (value of information or decision analysis).

At larger scales, remote sensing offers added possibilities. This can either be by relating UAV, airplane or satellite data directly to soil properties, or by inferring changes in SOC by vegetation changes, or by using remote imagery as a covariate in digital soil mapping of SOC. Direct interpretation can be performed on hyperspectral imagery in combination with spectral libraries for direct quantification of bare soil patterns (top 1 cm; Gomez, Lagacherie, & Bacha, 2012; Jaber, Lant, & Al‐Qinna, 2011), or by using multivariate imagery for mapping bare soil patterns as indication of SOC or soil class differences either using raw or enhanced imagery such as by multi‐temporal composites (Gallo et al., 2018; Rogge et al., 2018).

Changes in vegetation patterns visible in remote imagery can be used to detect (changes in) land use and thus infer soil properties and SOC change. Analysis of land‐use change, net primary productivity and SOC stocks are instrumental for identifying hotspots of SOC sequestration potential (Caspari, Lynden, & Bai, 2015; van der Esch et al., 2017).

The third option is to use satellite imagery products as covariates in digital soil mapping, where the relation between soil properties and satellite information is used to predict SOC maps at various depths using point observations and satellite imagery products (Hengl et al., 2017; McBratney, Mendonça Santos, & Minasny, 2003; Minasny & McBratney, 2016).

Remote sensing offers a range of possibilities, detail and spatial scales that are not feasible with point measurements alone (Ge, Thomasson, & Sui, 2011; Mulder, Bruin, Schaepman, & Mayr, 2011). That said, a combination of remote and in situ or point data will remain necessary to derive high resolution and accurate SOC maps. Apart from the limited penetration depth (top 1 cm while a soil profile would be desirable), this is also due to the fact that in many regions, bare soil is never visible, or areas are too often covered in clouds. At the same time, the high temporal frequency and high spatial resolution of remote imagery offer an unprecedented possibility to study and monitor space–time dynamics of SOC change if used in combination with (long‐term) monitoring stations (Chabrillat et al., 2019).

## 4 REPEATED SOIL SURVEYS—NATIONAL/SUB NATIONAL

Repeat soil sampling programmes have been conducted in a number of countries, such as England and Wales (Bellamy, Loveland, Bradley, Lark, & Kirk, 2005; Kirkby et al., 2005), Denmark (Heidmann, Christensen, & Olesen, 2002; Taghizadeh‐Toosi, Olesen, et al., 2014), Belgium (Sleutel, Neve, & Hofman, 2003) and New Zealand (Schipper et al., 2014—see below). These rely on resampling of previously sampled locations after varying periods. Advantages are that repeat sampling schemes measure actual soil carbon contents over large spatial scales and over long periods (Bellamy et al., 2005), but the main disadvantage is that land‐use change and land management between sampling periods are mostly unknown, making attribution of any observed changes in soil carbon to specific drivers (such as management or climate change) very difficult (Smith et al., 2007). In some cases, records of land use and management have been available allowing the effect of management changes to be assessed for better verification of modelling approaches to quantifying SOC stock changes (Taghizadeh‐Toosi, Olesen, et al., 2014).

Resampling of soil survey sites originally sampled in the 1970s–1990s in New Zealand has played an important role in identifying changes in soil carbon stocks in grazed pastures (Schipper et al., 2014). The difficulty with these historical resampling efforts was that sites were not chosen with national survey purposes in mind, so their representativeness was questionable. Additionally, sampling efforts were not carried out uniformly over space and time, so resampling was potentially confounded by the effects of soil type, climate and other factors. However, these data have been central to development and subsequent implementation of more robust sampling designs of grazed lands. Alongside, resampling of site impacts of management practices on carbon stock has been explored through the sampling of adjacent long‐term management practices (e.g. Barnett, Schipper, Taylor, Balks, & Mudge, 2014; Mudge et al., 2017).

In the case of Europe, differences exist in the availability of soil surveys among countries. As highlighted in the final report of the ENVASSO project, soil monitoring networks are much denser in northern and eastern European countries compared with countries located in the southern part of the continent (Kibblewhite et al., 2008). For example, countries such as France, Sweden or Poland maintain systematic soil monitoring systems at national level with different density of monitoring sites and sampling frequencies. In the case of France, different soil monitoring system levels exist which operates to either forest and non‐forest areas. The Soil Quality Monitoring Network was created 20 years ago for non‐forested areas, covering the main land uses in France in a 16 × 16 km grid (King, Stengel, Jamagne, Le Bas, & Arrouays, 2005). Similarly, in Sweden, soil monitoring is performed at two geographical levels (national and regional) and with different levels of application: forest land, integrated monitoring (areas with minor impact of forest management), intensive monitoring plots (223 forest plots) and arable land monitoring (Olsson, 2005). Poland has also different soil monitoring systems for forest and cropland soils. For the case of croplands, monitoring soils started in 1994 and since then soils have been sampled every 8 years with different soils' properties measured (Białousz, Marcinek, Stuczyński, & Turski, 2005). In Denmark, soils are sampled every 8–10 years to 1 m depth on a regular 7 km grid covering both agricultural and forest soils (Taghizadeh‐Toosi, Olesen, et al., 2014).

In contrast, EU Mediterranean countries such as Italy, Spain or Greece are examples of European regions where systematic national soil monitoring systems are underdeveloped or non‐existent, despite the risks of SOC losses, and soil erosion events resulting from a combination of crop management and regional impacts of climate change (Trnka et al., 2011). For example, in the case of Italy, there is no monitoring system, but there is willingness to develop it. In Spain, over the last 20 years, two independent soil national inventories have been performed; one to assess soil erosion and the other to asses soil heavy metal pollution (Ibáñez, Sánchez Díaz, de Alba, López Arias, & Bioxadera, 2005). However, the inventories have not been linked and there is no firm schedule for future resampling yet in place.

## 5 LONG‐TERM EXPERIMENTS OF SOC CHANGE

Since changes in bulk soil carbon occur slowly (Smith, 2004a), long‐term measurements are required to show the relatively small change against the large background carbon stock. To this end, long‐term field experiments exist in various parts of the world, with some dating from the 19th century. Although many of these experiments were originally set up to examine the effects of management (often fertilization) on crop or grass yield, many have a history of measurements of soil carbon and nitrogen change. Over recent decades, results from these field experiments have been central to testing the accuracy of models of turnover of SOC. As noted by Smith et al. (2012), the long‐term experiments in various parts of the world existed largely in isolation of each other, but in the 1990s, there were attempts to bring the various experiments together into shared networks (Barnett, Payne, & Steiner, 1995), with two such networks focussing on soil C; the Soil Organic Matter Network (SOMNET) and EuroSOMNET (the more detailed European component of the larger global network) were two attempts to couple SOC models with observations from long‐term experiments (Smith et al., 1997), with the aims or both testing models and the sharing, comparing and use of data from across the experiments to estimate carbon sequestration potential (Smith, Powlson, Smith, Falloon, & Coleman, 2000). SOMNET later evolved into an online, real‐time inventory project with a website known as Long‐Term Soil‐Ecosystems Experiments, which now has collected metadata on well over 200 long‐term soil experiments Richter, Hofmockel, Callaham, Powlson, and Smith (2007), with the metadata currently hosted by the International Soil Carbon Network (http://iscn.fluxdata.org/partner-networks/long-term-soil-experiments/). Smith et al. (2012) showed the locations and purpose of these long‐term experiments. Most (>80%) of the world's long‐term field studies address agricultural research questions, and most of the field studies test agricultural questions in the temperate zone. Nonagricultural sites and experiments in the bioclimatic zones other than the temperate region are under‐represented (Smith et al., 2012).

Long‐term field studies have proved extremely valuable for understanding the long‐term dynamics of SOC and wider issues of soil sustainability (Richter et al., 2007). In terms of MRV, the long‐term experiments serve as (a) a long‐term record of change; (b) a test bed for SOC models; (c) locations where new practices could be tested and measured; and (d) sites where shorter term (e.g. flux measurements) could be taken to better understand shorter term processes. Such experiments could therefore form vital components of national and international MRV platforms for SOC change. Existing long‐term monitoring sites are extremely valuable but do not exist in every global region, making a compelling case for starting new long‐term experimental/ monitoring sites in under‐represented regions.

## 6 MODELS OF SOC CHANGE

The soil organic matter (SOM) dynamics can be described by different mathematical formulations (Parton, Grosso, Plante, Adair, & Luz, 2015), as presented in Table 1, and different model approaches (Campbell & Paustian, 2015; Manzoni & Porporato, 2009). Most common SOM models are compartment models, which use between two and five carbon pools (Falloon & Smith, 2000). While the stability and complexity of the organic compounds is not represented explicitly in models, it is represented by varying turnover and residence times of OC in different carbon pools (Stockmann et al., 2013). The residence times are controlled by the decay rate of the carbon in the different pools, which is usually described by the first‐order kinetics (e.g. Falloon & Smith, 2000; Parton et al., 2015; Paustian, 1994). A wide range of different models show this structure, either as independent SOM model or as part of an ecosystem model, dynamic vegetation model or a general circulation model (Campbell & Paustian, 2015; Ostle et al., 2009; Parton et al., 2015). Manzoni and Porporato (2009) identified about 250 different models, but there are still new developments, as there are still unresolved challenges.

**Table 1:** List of different functions to simulate the decomposition in models following the discussion of Parton et al. (2015). The publications listed refer to the example models. The abbreviations describe the carbon (C) at the start (C_0_) and at a certain time (*t*) step (C*_t_*), the decomposition rate (*k*), the Michaelis–Menten constant (*K*_m_) and the maximum reaction velocity for the process (*V*_m_), the carbon demand by the microbes (*X*_0_), the Monod constant (*K*_*t*_) and the maximum growth rate (*µ*_max_). The graphs show C*_t_* in a time series for one set of arbitrary parameters

Approach	Equation	Graphical relation (C(*t*))	Example model	Publications
Zero‐order kinetics	$C_{t}=C_{0}-kt$	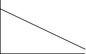		
First‐order kinetics	$C_{t}=C_{0}e^{-kt}$	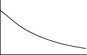	RothC, ICBM	Jenkinson and Rayner (1977), Andrén and Kätterer (1997)
Enzyme kinetics	$\frac{\text{dC}}{dt}=V_{m}\frac{C}{K_{m}+C}$	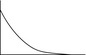	CLM, SEAM	Wieder et al. (2013), Wutzler et al. (2017)
Microbial growth	$-\frac{\text{dC}}{dt}=\mu_{\max}\left(\frac{C}{K_{t}+C}\right)\left(C_{0}+X_{0}-C\right)$	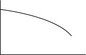	NICA	Blagodatsky and Richter (1998)

Despite the development of different approaches that allow the measurement of different carbon pools in the models (e.g. Janik et al., 2007; Skjemstad, Spouncer, Cowie, & Swift, 2004; Zimmermann, Leifeld, Schmidt, Smith, & Fuhrer, 2007), SOC pools are often still initialized in a spin‐up run (Nemo et al., 2017). This is a practical approach if information about the fractionation is not available, but it relies on ideal assumptions of equilibrium (Smith, Smith, Monaghan, & MacDonald, 2002) which impacts the results (Bruun & Jensen, 2002). Furthermore, the residence times of most pools exceed the duration of available measurements, which makes the calibration and validation of the models difficult (Campbell & Paustian, 2015; Falloon & Smith, 2000). Additionally, not all relevant processes (e.g. priming) are represented in the models (Guenet, Moyano, Peylin, Ciais, & Janssens, 2016; Wutzler & Reichstein, 2013). Recently, there has been a discussion about the ability of existing models to reflect changes in temperature (Conant et al., 2011; Moyano, Vasilyeva, & Menichetti, 2018), which is most relevant to simulate climate change impacts (Conant et al., 2011). In short, it is not clear, if the slower, more stable pools get differently affected by temperature changes (e.g. Campbell & Paustian, 2015; Conant et al., 2011). For these and other purposes, there are an increasing number of new model approaches and hypotheses (e.g. Cotrufo, Wallenstein, Boot, Denef, & Paul, 2013; Lehmann & Kleber, 2015; Wieder, Bonan, & Allison, 2013; Wutzler, Zaehle, Schrumpf, Ahrens, & Reichstein, 2017). Therefore, long‐term data sets (Section 5) are needed to test the performance of the established and the new models.

Many operational SOC models only simulate turnover and decomposition of the SOC pools and the added OC (Toudert et al., 2018). These models thus rely heavy on proper estimation of carbon inputs in residues and organic amendments (manure, compost, etc.) as well as on information on the biological quality of these inputs. Most modelling approaches used for inventory purposes rely on input data from harvest residues or decaying plant parts and external organic amendments. The plant C inputs are mostly derived from measured agricultural yields using simple allometric equations, where the C inputs is related linearly or non linearly to crop yield (Keel, Leifeld, & Mayer, 2017). Comparison of different published approaches of estimating C input, but using the same decomposition model, has demonstrated large uncertainties in simulated changes in SOC (Keel et al., 2017). The selection of allometric functions for estimating C input is therefore a critical step in the choice of model approach. Recent research has also questioned the appropriateness of using simple allometric functions such as fixed shoot:root ratios for estimating C input (e.g. Hu et al., 2018). Rather than assuming a fixed shoot:root ratio, using a fixed amount of belowground C input depending on site and crop may provide the most robust estimate (Hirte, Leifeld, Abiven, Oberholzer, & Mayer, 2018; Taghizadeh‐Toosi, Christensen, Glendining, & Olesen, 2016). This has implications for modelling application where changes in crop productivity are a main driver of C inputs.

## 7 WHAT MRV PLATFORMS ARE CURRENTLY IN USE

A number of GHG emission and soil carbon change quantification schemes have been developed in various parts of the world. For example, the Australian Carbon Farming Initiative/Emission reduction fund has guidance relating to sampling and measurement of SOC and estimating and reporting SOC stock change for SOC management projects (Australian Government, 2018). In Alberta in Canada, there is a Conservation Cropping Protocol, a tool used to quantify GHG emission reductions from conservation cropping (Alberta Government, 2012). For certain production systems (e.g. livestock production), FAO has published guidance on measuring and modelling soil carbon stocks and stock changes (FAO, 2019a). In this section, we examine methods already in use in countries participating in the Global Research Alliance of Agricultural Greenhouse Gases (GRA).

### 7.1 Operational soil MRV systems in use in GRA countries

We first searched the GRA publications library (https://globalresearchalliance.org/publication-library/) for operational soil MRV systems/procedures, giving limited results (e.g. Minamikawa, Yamaguchi, Tokida, Sudo, & Yagi, 2018). Subsequently, we searched the Web‐of‐Science using “((soil AND carbon) OR soc) AND ((monitoring OR reporting OR verification) OR mrv),” giving 91 potential sources. Adding the GRA country names (56 as of October 2018) to the initial search reduced this to 14 papers. These studies cover parts of a country (McHenry, 2009; Nerger, Funk, Cordsen, & Fohrer, 2017; Steinmann et al., 2016; Wilson, Barnes, Koen, Ghosh, & King, 2010), consider selected agro‐ecosystems or agricultural practices (Allen, Pringle, Page, & Dalal, 2010; de Gruijter et al., 2016; McHenry, 2009; Scott et al., 2002; Wu, Clarke, & Mulder, 2010), outline the basis for a possible national soil monitoring system (Spencer, Ogle, Breidt, Goebel, & Paustian, 2011; Visschers, Finke, & Gruijter, 2007), were discontinued due to lack of funding (Goidts, Wesemael, & Oost, 2009; Taghizadeh‐Toosi, Olesen, et al., 2014; Yagasaki & Shirato, 2014) or, alternatively, concern measurement systems that are in their first (Mäkipää, Liski, Guendehou, Malimbwi, & Kaaya, 2002; Nijbroek et al., 2018) or second round (Orgiazzi, Ballabio, Panagos, Jones, & Fernández‐Ugalde, 2018; Spencer et al., 2011).

Much early work has been done in Australia (McKenzie, Henderson, & Mcdonald, 2002), and in 2014, the Australian Government approved the first methodology for soil carbon sequestration for use at farm level (de Gruijter et al., 2016); recommended procedures of stratification and sampling, however, may vary between countries (e.g. Australia and New Zealand, see Malone et al., 2018). Overall, a lack of common procedures between (and within) countries affects the suitability of using the SOC stock as absolute indicator for monitoring changes in land quality and soil degradation, for example, in relation to the SDG monitoring framework (Sims et al., 2019). Earlier reviews (Batjes & van Wesemael, 2015; de Brogniez, Mayaux, & Montanarella, 2011; Lorenz, Lal, & Ehlers, 2019) also indicated that basic soil data and SOC stock change monitoring systems are not available, or inconsistent (Jandl et al., 2014), for many regions and nations. Within the GRA and the CGIAR CCAFS programme, the initial focus has been on MRV resources for the livestock sector (Wilkes, Reisinger, Wollenberg, Van, & Dijk, 2017).

There are three main approaches (experimental field trials, chronosequence studies or paired land‐use comparisons, and monitoring networks) to determine relationships between environmental and management factors, and SOC dynamics and GHG emissions (Batjes & van Wesemael, 2015; McKenzie et al., 2002; Morvan et al., 2008; Spencer et al., 2011) or changes in soil quality/health (Bai et al., 2018; Leeuwen et al., 2017). An overview of long‐term terrestrial soil experiments (LTEs) is maintained by the International Soil Carbon Network, including those from a European Network of long‐term studies for soil organic matter (SOMNET, Powlson et al., 1998). Examples of chronosequence studies include those carried out in Brazil (Cerri et al., 2007; de Moraes Sá et al., 2009), Ethiopia (Lemenih, Karltun, & Olsson, 2005) and China (He, Wu, Wang, & Han, 2009), while paired land‐use comparisons have been reviewed by various researchers (Bai et al., 2018; Murphy, Rawson, Ravenscroft, Rankin, & Millard, 2003; Oliver et al., 2004).

Following up from the review of European soil monitoring networks (Morvan et al., 2008), the Joint Research Centre of the European Commission launched an initiative to sample the topsoil at 22,000 points of the Land Use/Cover Area Survey (LUCAS project, see Montanarella, Tóth, & Jones, 2011). The first soil sampling round (2009), based on standard sampling and analytical procedures, followed a stratified sampling design to produce representative soil samples for major landforms and types of land cover of the participating countries. A new LUCAS sampling round is presently underway, providing the basis for a longer term monitoring system (Orgiazzi et al., 2018). Similarly, for the United States, Spencer et al. (2011) discuss the design of a national soil monitoring network for carbon on agricultural lands, including determination of sample size, allocation and site‐scale plot design. Teng et al. (2014) indicated that for accurate soil monitoring in China, it will be necessary to set up routine monitoring systems at various scales (national, provincial and local scales), taking into consideration monitoring indicators and quality assurance.

Table 2 serves to illustrate the diversity in soil monitoring networks and sample designs in selected GRA countries. The most common sampling design for networks aimed at monitoring regional/national SOC stocks is either stratified (according to soil/land use/climate) or grid based. Large countries with a low sampling density (<1 site per 100 km^2^) generally adopt a stratified design to include all important units (van Wesemael et al., 2011). The (expected) variability within these units should be determined to assess the optimal number of samples for each stratum (Brus & de Gruijter, 1997; De Gruijter, Brus, Bierkens, & Knottters, 2006; Louis et al., 2014). Such an approach will allow a (geo)statistical analysis of SOC stock changes for the soil/land use/climate units under consideration as an alternative or test for process‐based models. Continuous soil monitoring for SOC at time intervals of 10 year is often proposed as a compromise between minimum detectability of changes (Garten & Wullschleger, 1999) and temporal shifts in trends (Bellamy et al., 2005; Schrumpf, Schulze, Kaiser, & Schumacher, 2011; Steinmann et al., 2016). This may be longer than the duration of many land‐use management projects that involve the measurement of SOC stock changes (Milne et al., 2012).

**Table 2:** Examples of soil monitoring networks and sample design in selected GRA countries^a^

	Belgium	Brazil	China	Mexico	New Zealand	Sweden
Objective	National SOC monitoring	SOC response to land use/management change	Regional SOC monitoring	National SOC monitoring	National SOC monitoring	National SOC monitoring
Region covered	Cropland and grassland in southern Belgium	Rodônia, Mato Grosso, Central Amazonia	Northeast (120 sites), North (241), East (356), South (119), Northwest (148), Southwest (97)	Forest and non‐forest land in particular pasture and shrubs	All regions and land uses	Cropland~3 Mha
Starting date	National Soil Survey 1950–1970; resampled 2004–2007	~2007	78% started before 1985 and 87.5% continued until at least 1996	Started in 2003; each year one‐fifth of the sites will be resampled	National soils database from 1938; Land use and carbon analysis system started in 1996^c^	Full scale in 1995, some data from 1988
Site density (km^2^ per site)	18 km^2^	N/A	N/A	78 km^2^	202 km^2^	10 km^2^
Site selection	Stratified	Stratified	Stratified	Grid	Stratified	Grid
Soil sampling						
Subsamples	Composite	Composite	Composite	Composite	Single	Composite
Depth	0–30 and 0–100 cm	0–10, 10–20, 20–30, and 30–40 cm	0–20 cm	0–30 and 30−60 cm	Variable, sampled by soil horizon; in 2009, 1,235 samples to 30 cm	0–20 and 40–60 cm
Frequency	Future sampling rounds largely depend on funding (Goidts et al., 2009)	Once (chronosequences and paired sites)	Annual sampling from 2010, see Teng et al. (2014)^b^	Every 5 years	A fit‐for‐purpose method is being designed to monitor SOC stocks at ~5 year intervals over upcoming decades	1995 and 2005 round completed; in principle repeated every 10 years

Abbreviation: SOC, soil organic carbon.

a Adapted from Van Wesemael et al. (2011).

b For accurate soil monitoring in China, it will be necessary to set up routine monitoring systems at various scales (national, provincial and local scales), taking into consideration monitoring indicators and quality assurance (Teng et al., 2014).

c For recent developments, see https://soils.landcareresearch.co.nz/index.php/soils-at-manaaki-whenua/our-projects/soil-organic-carbon.

New Zealand has developed a model‐based approach (McNeill, Golubiewski, & Barringer, 2014; Tate et al., 2005) to track SOC stock changes with time assuming that SOC stock values vary by soil type, climate and land use, and that the key driver for long‐term (decadal) changes in SOC stocks are due to changes in land use, with all other changes due to soil, climate or erosion assumed constant. This country‐specific (Tier 2) empirical method was initially described in Tate et al. (2005) reflecting land‐use change issues relevant to New Zealand. As further soil profile data were collected (currently 2050 profiles) the model was increasingly improved (McNeill et al., 2014) adding data from specific land‐use classes (notably indigenous and exotic forest, cropland, horticulture and wetlands). The approach was also refined to account for spatial autocorrelation to improve the assessment of the overall significance of land‐use change and reports three validation studies for the model (McNeill et al., 2014). Using low‐producing grassland on a high‐activity clay IPCC default soil and moist‐temperate IPCC default climate class as a reference, the 0–30 cm SOC stock is 133.1 tonnes/ha, the change as a result of land use can be determined, along with the marginal significance. For example, a transition to high‐producing grassland results in a change of −0.22 tonnes/ha (not significant), while a transition to perennial cropland results in a change of −19.5 tonnes/ha (significant).

While changes in national or large regional scale carbon stock measurements can be addressed using geostatistical sampling approaches, aligned targeted approaches (such as sampling of chronosequences and paired land uses) can directly determine land‐use change factors, while controlling for other spatially dependent variables, that is, they can determine the carbon gain/loss that will occur with a change in land use or management. When coupled with monitored changes in land area undergoing these changes, estimates of national scale carbon stock changes can be calculated. The change in carbon stocks determined from paired site sampling can also be used to validate interpretations derived from national scale measurements.

### 7.2 Methods used by GRA countries for estimating SOC changes for the ‘cropland remaining cropland’ category in national inventories

All countries that are party to the United Nations Framework Convention on Climate Change (UNFCCC) are required to provide national inventories of emissions and removals of GHG due to human activities. The IPCC methodologies are intended to yield national GHG inventories that are transparent, complete, accurate, consistent over time and comparable across countries. Because different countries have different capacities to produce inventories, the guidelines lay out tiers of methods for each emissions source, with higher tiers being more complex and/or resource intensive than lower tiers. In the context of agricultural GHG emissions, inventories remain the main tool connecting policy with mitigation.

**Figure 2:**
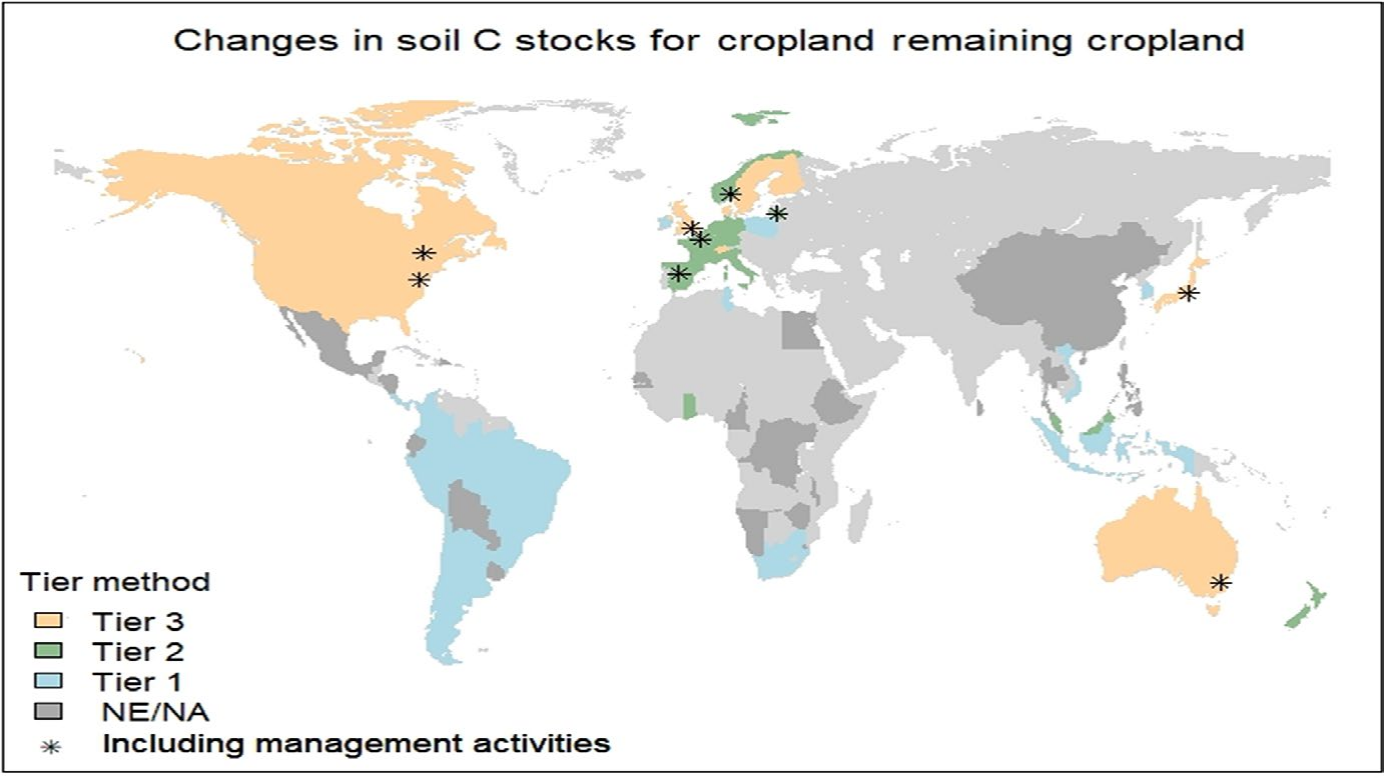
Tier methods used by Global Research Alliance of Agricultural Greenhouse Gases countries for estimating the changes in mineral soil carbon stock for the ‘Cropland remaining Cropland’ category. NA indicates that the country has not developed a GHG inventory. NE indicates that the country has not included soil organic carbon changes in croplands in the inventory. Countries reporting carbon stock change associated with agricultural land use and management activities are indicated by (*)

Figure 2 shows the categories of methods used by GRA countries for estimating the changes in mineral soil carbon stock for the ‘Cropland remaining Cropland’ category. Countries listed as non‐annex I face major challenges with either non‐existent data (15 countries do not have country‐specific information they can use to develop their inventory and eight countries do not consider for SOC changes in croplands because do not have the technical capacity to monitor these sources) or a lack of relevant data (with the exception of Ghana and Malaysia) GRA non‐annex I countries use a Tier 1 approach to report SOC changes associated with areas defined as Cropland land use.

Soil C stocks are influenced by multiple factors that affect primary production and decomposition, including changes in land use and management and feedbacks between management activities, climate and soils. However, only a few countries have taken into account cropland management activities. Table 3 provides an overview of the methods used in GRA countries for estimating carbon stock change and emissions associated with agricultural land use and management activities on mineral soil.

**Table 3:** Methodology used to estimate changes in soil C stocks for cropland remaining cropland, including agricultural land use and management activities on mineral soils

GRA country	Tier	Land management activities	Reference
Australia			
The Full Carbon Accounting Model (FullCAM)	Tier 3	Crop type and rotation (including pasture leys)	Richards (2001)
		Stubble management, including burning practices	
		Tillage techniques	
		Fertilizer application and irrigation	
		Application of green manures (particularly legume crops)	
		Soil ameliorants (application of manure, compost or biochar)	
		Changes in land use from grassland	
Crop‐specific coefficients sourced from the literature combined with ABS agricultural commodities statistics	Tier 2	Changes in the area of perennial woody crops	
Canada			
Process model (CENTURY) based on the National Soil Database of the Canadian Soil Information System	Tier 3	Change in mixture of crop type (increase in perennial crops and increase in annual crops)	McConkey et al. (2014)
		Change in tillage practices	
		Change in area of summer fallow	
		Land use, tillage, type and amount of input	
		Crop residue, farmyard manure and presence or absence of vegetative cover	
		Perennial and organic management systems	
Denmark			
Average SOC calculated annually per soil type and region based on process‐based model (C‐TOOL) using data on temperature and estimated C input from crop residues and manure using national databases	Tier 3	Crop type and crop yield	Taghizadeh‐Toosi and Olesen (2016)
		Cover crops	
		Residue management	
		Manure application	
		Grassland management	
France			
The IPCC Guidelines and OMINEA database	Tier 1	Land use	CITEPA (2019)
		Tillage	
		Type and amount of input	
Japan			
Average carbon stock changes in each year by land‐use subcategory (rice fields, upland fields, orchards and pastural land) calculated by the Roth C model by the mineral soil area of each prefecture obtained from statistical material, map data and questionnaire survey	Tier 2	Carbon input from crop residue	Shirato and Taniyama (2003)
		Farmyard manure	
		Presence or absence of vegetative cover	
Lithuania			
National statistics for woody crops and available data of arable land certified as organic in FAOSTAT and ecological agricultural land statistics.	Tier 2	Crop type (perennial crops, certified organic crops, other crops)	Statistics Lithuania (2018)
		Amount of input	
Norway			
Reference stock and stock change factors estimated by the Introductory Carbon Balance Model (ICBM) in a study where CO_2_ emissions were estimated for Norwegian cropland	Tier 2	Crop rotations	Borgen et al. (2012)
		Carbon inputs	
		Tillage	
Spain			
SOC values calculated by use and province, together with the reference values of the management factors provided by the IPCC Guidelines	Tier 1	Land use	Rovira et al. (2007)
		Crop rotations	
		Amount of input	
		Tillage	
United Kingdom			
Review UK relevant literature on the effects of cropland management practices on soil carbon stocks to model UK‐specific emission factors (methodology developed in Defra project SP1113)	Tier 1	Manure	Moxley et al. (2014)
		Residue inputs	
		Crop type (perennial, cropland, set‐aside)	
	Tier 2	Tillage	
United States			
Published literature to determine the impact of management practices on SOC storage. Activity data based on the historical land use/management patterns recorded in the 2012 NRI (USDA, 2018)	Tier 2	Tillage	Ogle, Breidt, Eve, and Paustian (2003); Ogle, Breidt, and Paustian (2006)
		Cropping rotations	
		Intensification	
		Land‐use change between cultivated and uncultivated conditions	

Abbreviation: ABS, Australian Bureau of Statistics; GRA, Global Research Alliance of Agricultural Greenhouse Gases.

There are still high levels of uncertainty in the estimates; however, uncertainties are relatively low for Annex I countries due to their well‐developed statistical systems and capacity to use higher tier methods. In contrast, national inventories of many developing countries generally have greater uncertainty and are not sufficiently rigorous to enable monitoring of emissions. For Tier 2 inventory development, countries could use the expertise of other GRA members, for instance from those countries that have adopted a Tier 3 method (see Table 4) to estimate soil organic C stock changes in agricultural land.

**Table 4:** Models used to estimate carbon dioxide emissions and removals from the cropland remaining cropland soils component (Tier 3 method) in GRA countries

GRA country	Model		Reference
Australia	The Full Carbon Accounting Model (FullCAM)	Estimates emissions from soil through a process involving all on‐site carbon pools (living biomass, dead organic matter and soil) on a pixel by pixel (25 m × 25 m) level	Richards (2001)
Canada	CENTURY	Process model used for estimating CO_2_ emissions and removals as influenced by management activities, based on the National Soil Database of the Canadian Soil Information System	Parton, Schimel, Cole, and Ojima (1987), Parton, Stewart, and Cole (1988)
Denmark	C‐TOOL	3‐Pool dynamic soil model parameterized and validated against long‐term field experiments (100–150 years) conducted in Denmark, United Kingdom (Rothamsted) and Sweden and is ‘State‐of‐the‐art’	Taghizadeh‐Toosi, Christensen, et al. (2014)
Finland	Yasso07 soil carbon model	The parameterization of Yasso07 used in cropland was the one reported in Tuomi, Rasinmäki, Repo, Vanhala, and Liski (2011)	Palosuo, Heikkinen, and Regina (2015)
Japan	Soil Carbon RothC model	In order to apply the model to Japanese agricultural conditions, the model was tested against long‐term experimental data sets in Japanese agricultural lands (Shirato & Taniyama, 2003)	Coleman et al. (1997), Coleman, and Jenkinson (1987)
Sweden	Soil Carbon model ICBM‐region	Calculate annual C balance of the soil based on national agricultural crop yield and manure statistics, and uses allometric functions to estimate the annual C inputs to soil from crop residues	Andrén and Kätterer (2001)
Switzerland	Soil Carbon RothC model	The implementation of RothC in the Swiss GHG inventory is described in detail in Wüst‐Galley, Keel, and Leifeld (2019)	Coleman et al. (1997), Coleman and Jenkinson (1987)
United Kingdom	CARBINE Soil Carbon Accounting model (CARBINE‐SCA)	Simplified version of the ECOSSE model (Smith, Gottschalk et al., 2010), coupled with a litter decomposition model derived from the ForClim‐D model (Liski, Perruchoud, & Karjalainen, 2002; Perruchoud, Joos, Fischlin, Hajdas, & Bonani, 1999)	Matthews et al. (2014)
United States	DAYCENT biogeochemical model	Utilizes the soil C modelling framework developed in the Century model (Parton et al., 1987, 1988, 1994; Metherell, 1993), but has been refined to simulate dynamics at a daily time step	Parton, Hartman, Ojima, and Schimel (1998), Del Grosso et al. (2001), Del Grosso and Parton (2011)

Abbreviation: GRA, Global Research Alliance of Agricultural Greenhouse Gases.

With increased obligations for reporting on GHG emissions and Nationally Determined Contributions (NDCs) under the Paris agreement, it is important that all countries are able to estimate their GHG emissions to maximize transparency, accuracy, completeness and consistency. Improving inventories requires enhanced national capability to gather relevant activity data to develop country‐specific emission factors. There is a need to improve the evidence base and to better connect governments and relevant expertise to subsequently improve the quality of agricultural NDCs and the way their achievements are reflected by national GHG inventories.

## 8 PROPOSED GLOBAL SOIL MRV PLATFORM

**Figure 3:**
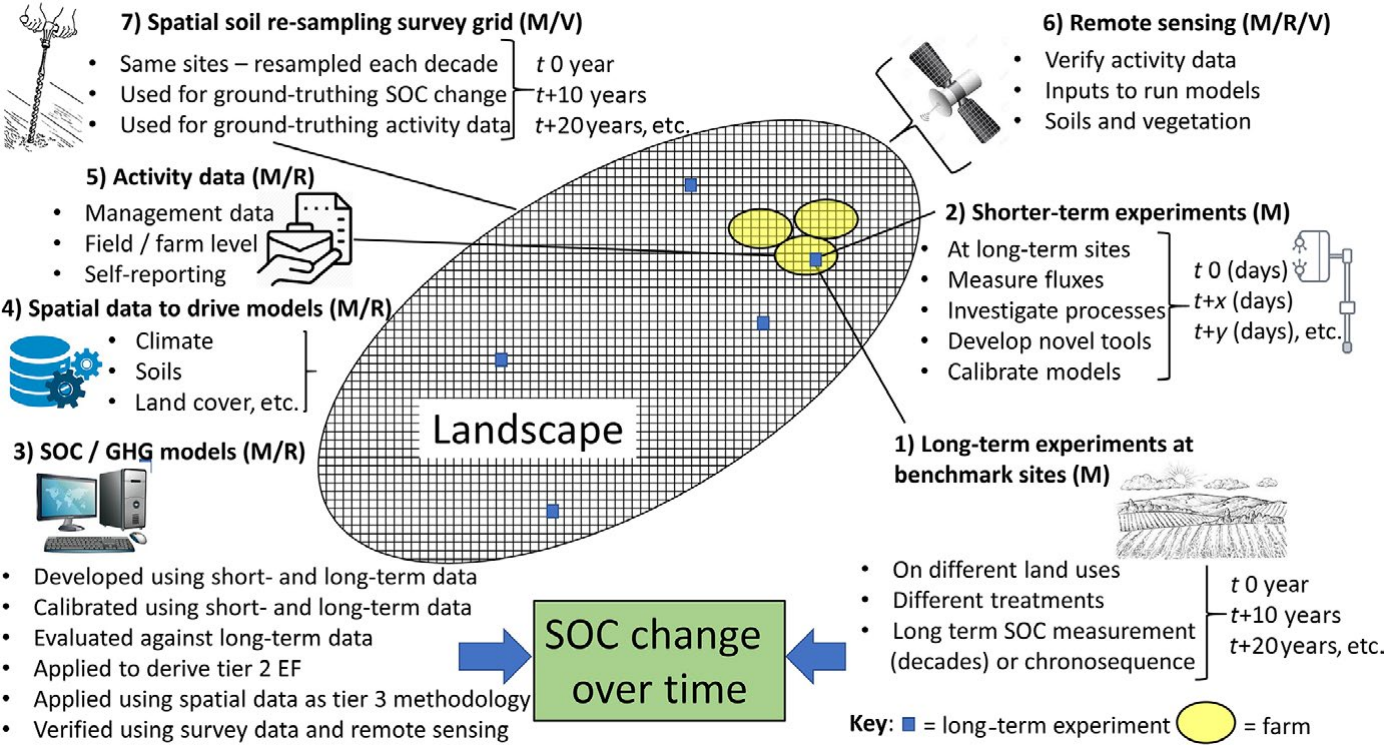
Components of a soil measurement/monitoring, reporting and verification framework, indicating which components contribute to measurement/monitoring (M), reporting (R) or verification (V). See text in Section 8 for explanation of linkages between the components

The sections above describe the methods available to measure and monitor carbon; models that can be used to simulate and project changes in SOC, different types of experimental platform and the data needed to test models and allow them to be run from plot to global scale; and methods/platforms that could be used to verify any simulated change in SOC (summarized in Figure 3). These form the components of a system suitable for MRV of SOC change (Figure 3).

Central to the system are benchmark sites, which could be located at existing or new long‐term experiments (Figure 3, item 2; Richter et al., 2007), or could consist of well‐characterized chronosequences or paired sampling sites (e.g. He et al., 2009; Oliver et al., 2004). The benchmark sites would preferably be located on representative land cover/land‐use types, soil types and with representative management. At these sites, proposed practices to increase SOC could be tested in fully randomized block designs, and SOC change measured over time (measurements every few years), while measuring shorter term processes (such as GHG emissions) more frequently (continuously with EC flux towers or frequently with automated chambers; Figure 3, item 2; Baldocchi, 2003). The same sites could be used to test novel spectral methods for measuring SOC change against traditional direct SOC measurement (England & Viscarra Rossel, 2018). Careful alignment of site selection and experimental design with other goals of land owners, managers and regulators (e.g. quantification of soil quality change or nutrient use efficiency) will promote stronger uptake of an international suite of benchmark sites with additional benefits.

Since it would be prohibitively expensive to set up benchmark sites covering all possible combinations of land use, climate, soil type and management practice, models of SOC change are required to interpolate and infer change across all combinations, and to project changes into the future, across landscapes and under novel combinations (Figure 3, item 3; e.g. Richards et al., 2017). To establish confidence that the chosen model or models are capable of accurately and reliably simulating SOC change, they need to be tested across the full range of parameter space (i.e. multiple soils types, climate zones, land‐use types and soil management options; Ehrhardt et al., 2018; Smith et al., 1997). If necessary, the models can be further developed or parameterized using data from the same long‐term experiments, or from shorter term experiments, before being evaluated again against a data set not used in development or parameterization (Smith & Smith, 2007).

When the model(s) are deemed to be reliable, they could be applied (a) to derive IPCC Tier 2 emission or SOC stock change factors, which are specific to the region and conditions represented within the region (e.g. Begum et al., 2018); or (b) spatially over the whole landscape (or the entire land area of a country) using spatial databases of soil characteristics, and land cover, management and climate data (Figure 3, item 4), to directly simulate SOC change and GHG emissions, thereby delivering a Tier 3 methodology to report emissions (Smith et al., 2012). Data on changes in soil management are necessary for estimating changes in SOC/GHG emissions, and this could also be provided by self‐reported or farm survey‐derived activity data (Figure 3, item 5).

If self‐reported activity data are used as the primary mechanism for reporting, such activity data could be verified through spot checks/ farm visits or could be done using remote sensing (Figure 3, item 7), which can show, for example, the presence of bare fallow, cover crop or residue retention (Gallo et al., 2018; Rogge et al., 2018). In addition to providing a mechanism for verification of activity data, remotely sensed earth observation products could also provide spatial data to run the SOC/ GHG models. For example, earth observation can be used to estimate changes in carbon input to soils, through changes in NPP/GPP (Chen et al., 2019; Neumann & Smith, 2018), land degradation (Sims et al., 2019) and can also be used to determine land cover/land cover change (e.g. Chen et al., 2019).

Well‐calibrated models, supported by measurements, can also be used to establish relationships between a management change in a particular situation (combination or soil, climate, land use and management) and a change in SOC/ GHG emissions, including estimates of uncertainty (Fitton et al., 2017). This would allow activity data (Figure 3, item 5), self‐reported by the farmer/land manager, to be used as the primary source of data for reporting, in place of the need to directly measure SOC of GHG emission change (Smith, 2004b). More broadly, uncertainties and potential biases in all components of the MRV framework, including all measurements and modelling schemes, need to be addressed. For transparency, there is a need for unified protocols for such uncertainty assessments.

In terms of verification, change in SOC stocks, spatial soil monitoring networks (Figure 3, item 6) could be used to ground‐truth SOC changes estimated by the Tier 2 method or Tier 3 model projections over time. If resampled every few years, the soil monitoring network (on a grid as shown in Figure 3 item 7, e.g. Bellamy et al., 2005, or using a stratified sampling protocol; Montanarella et al., 2011) could provide independent estimates of large‐scale SOC change. Some basic methodological requirements and recommendations can be formulated for ‘good SOC‐monitoring and MRV practice’ to support scientific and policy decisions (Batjes & van Wesemael, 2015; Desaules, Ammann, & Schwab, 2010; Morvan et al., 2008; Spencer et al., 2011). These include: (a) the provision of long‐term continuity and consistency under changing boundary conditions, such as biophysical site conditions, climate change, methodologies, socio‐economic setting and policy context; (b) adoption of a scientifically and politically (e.g. for GRA, UNFCCC, UNNCCD) appropriate spatial and temporal resolution for the measurements; (c) ensuring continuous quality assurance at all stages of the measurement and monitoring process; (d) measurement/observation and documentation of all potential drivers of SOC and GHG change; and (e) soil monitoring network‐collated, georeferenced samples archived and the associated (harmonized) data made accessible through distributed databases to enhance the value of the collated data for multiple uses. In addition to this, soil monitoring networks should be included in a broader cross‐method validation programme to ultimately permit spatially and temporally validated comparisons both within and between countries. An open‐access database, where short‐ or long‐term soil C measurements could be uploaded and shared (e.g. https://dataverse.org/ or an online collaborative platform as used in the CIRCASA project: https://www.circasa-project.eu/), would also be of great benefit for progressing a global MRV system.

As indicated, the implementation of soil monitoring networks poses several scientific, technical and operational challenges. From an operational point of view, to implement an integrated monitoring system, it will be crucial to overcome initialization costs and unequal access to monitoring technologies. For developing countries, this will require international cooperation, capacity building and technology transfer (de Brogniez et al., 2011), which could be facilitated within GRA, CCAFS and similar organizations, in synergy with relevant funding mechanisms, or via the recently established ‘GSOCseq’ programme of the UN FAO (FAO, 2019b).

While other components of a soil MRV framework could be added, the components outlined in Figure 3 could certainly fulfil all of the functions necessary for an MRV system. As seen in Sections 4, 7, the existing capacity in terms of existing benchmark sites, soil monitoring programmes and access to models in different countries varies greatly. While some countries are already using Tier 2 and 3 monitoring of soil C change, others have barely begun to build capacity. Recently, the UN FAO has established a programme called ‘GSOCseq’ (FAO, 2019b) which aims to build this capacity internationally. Programmes such as this could pave the way for making this proposed MRV framework a reality.
